# Prevalence and Molecular Characteristics of Bovine Respiratory Syncytial Virus in Beef Cattle in China

**DOI:** 10.3390/ani12243511

**Published:** 2022-12-12

**Authors:** Yiming Chang, Hua Yue, Cheng Tang

**Affiliations:** 1 College of Animal and Veterinary Sciences, Southwest Minzu University, Chengdu 610041, China; 2 Key Laboratory of Ministry of Education and Sichuan Province for Qinghai-Tibetan Plateau Animal Genetic Resource Reservation and Utilization, Chengdu 610041, China

**Keywords:** bovine respiratory syncytial virus, epidemic, molecular characteristics, China

## Abstract

**Simple Summary:**

Bovine respiratory syncytial virus (BRSV) is an important pathogen causing cattle respiratory disease; however, the prevalence and molecular characteristics of BRSV in China remain largely unknown. The purpose of this study was to investigate the prevalence and molecular characteristics of BRSV in beef cattle with BRDC in China, and the results showed that BRSV had a wide geographical distribution, and subgroup III strains were the dominant strains in China. The Chinese strains in this study showed a unique evolutionary trend based on phylogenetic analysis of the G and F genes and genomic sequences, which contributed to a better understanding of the prevalence and genetic evolution of BRSV.

**Abstract:**

Bovine respiratory syncytial virus (BRSV) is an important pathogen of the bovine respiratory disease complex (BRDC); however, its prevalence and molecular characteristics in China remain largely unknown. In this study, 788 nasal swabs from 51 beef cattle farms with BRDC outbreaks in 16 provinces and one municipality were collected from October 2020 to July 2022, and 18.65% (147/788) of samples from 23 farms across 11 provinces were detected as BRSV-positive by reverse transcription-insulated isothermal PCR (RT-iiPCR) assay. Further, 18 complete G gene sequences were classified into BRSV subgroup III, and 25 complete F gene sequences were obtained from 8 and 10 provinces. Compared to the known BRSV strains in GenBank, the G proteins and F proteins in this study shared several identical amino acid (aa) mutations. Moreover, five nearly complete genome sequences were obtained and clustered into a large branch with two America BRSV subgroup III strains (KU159366 and OM328114) rather than the sole Chinese strain (MT861050) but were located in an independent small branch. In conclusion, this study reveals that BRSV has a wide geographical distribution in China, and subgroup III strains, which have unique evolution characteristics, are the dominant strains. The results contribute to a better understanding of the prevalence and genetic evolution of BRSV.

## 1. Introduction

Bovine respiratory syncytial virus (BRSV) belongs to *Orthopneumovirus*, of the family *Pneumoviridae*, and is an important pathogen of bovine respiratory disease complex (BRDC) [1]. According to the phylogenetic analysis of G gene nucleotide sequences, BRSV can be divided into subgroups I-X. A previous study showed that different subgroup strains had antigenic differences and obvious geographical distribution characteristics [2,3,4]: subgroup I has been detected in Britain and Switzerland [5]; subgroup II has been detected in Belgium, France, Denmark, Sweden, Japan, and the Netherlands [6]; subgroup III has been detected in America, China, Turkey, and Brazil, etc. [7,8,9]; subgroup IV has been detected in Germany, Belgium, Denmark, America, and other European countries [10]; subgroups V and VI have been detected in France and Belgium [11]; subgroups VII and VIII have been detected in Italy and Croatia [3]; subgroup IX has been detected in Brazil [12]; and subgroup X has been detected in Japan [13].

At present, there are 13 complete genomic sequences of BRSV in GenBank, including a subgroup II strain (MG947594) from Sweden, three subgroup III strains (KU159366, OM328114, and MT861050) from America and China, four subgroup IV strains (AF295543, NC_038272, NC_001989, and AF092942) from America and Germany, four strains of subgroup X (OM965699, OM965701, OM965702, and OM965703) from Japan, and an unclassified strain (OP0201460) from Australia. The genome of BRSV is 13,416–15,151 bp in length, encoding eleven proteins, including the small hydrophobic protein (SH), fusion protein (F), attachment glycoprotein (G), nucleocapsid protein (N), RNA-dependent RNA polymerase (L), phosphoprotein (P), matrix protein (M), matrix protein 2-1 (M2-1), matrix protein 2-2 (M2-2), and two nonstructural proteins (NS1 and NS2). The F protein and G protein are the main surface glycoproteins. The F protein is involved in the adaptive immune response to stimulate the production of neutralizing antibodies and can promote the entry of viral particles into the host cells, as well as mediating the fusion of infected cells to form syncytium [14,15,16]. The G protein is a type II glycosylated transmembrane protein that is mainly involved in receptor binding and the adsorption process [17].

The existence of BRSV in China was first confirmed in 2009 [18], and a sero-prevalence survey on healthy cattle in 14 provinces showed that the BRSV sero-positive rate was 41.2–94.4%, which proved that BRSV had a high infection rate in China [19]. Recently, BRSV was detected with a 6.95% positivity rate in clinical samples of cattle with BRDC in Inner Mongolia [20], and the presence of BRSV was also found in Northeast China [8,21,22]. However, the prevalence and molecular characteristics of BRSV in China remain largely unknown. The purpose of this study was to investigate the prevalence and molecular characteristics of BRSV in beef cattle with BRDC in China.

## 2. Materials and Methods

### 2.1. Samples Collection

**Figure 1:**
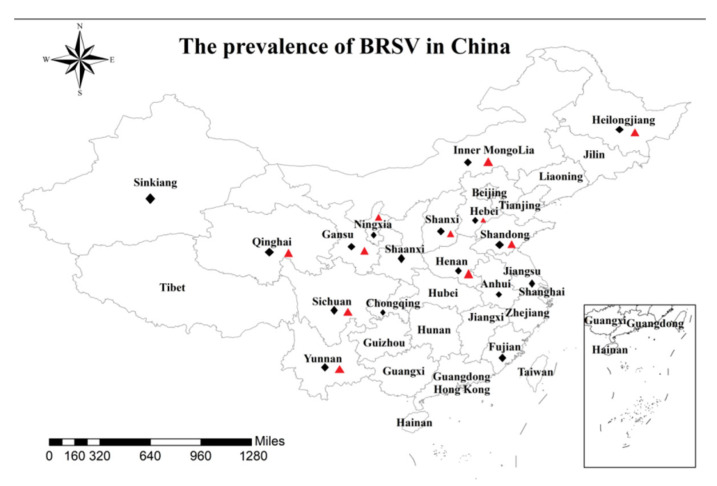
Map of China shows the geographical distribution of BRSV strains by province or municipality, 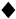 Indicates the province or municipality of the sample collected in this study, 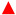 Indicates provinces with BRSV positive-sample distribution.

A total of 788 nasal swabs were collected from beef cattle with BRDC in 51 farms in 16 provinces and one municipality in China from October 2020 to July 2022, and the affected beef cattle were 2–6 months old. The sick beef cattle were characterized by runny nose, cough, dyspnea, etc. All samples were shipped on ice and stored at −80 °C in sterile 15 mL centrifuge tubes. Detailed information on samples is shown in Table 1 and Figure 1.

### 2.2. RNA Extraction and cDNA Synthesis

The nasal swabs were diluted 1:5 (*w*/*v*) with phosphate-buffered saline (PBS) and centrifuged at 10,000× *g* for 8 min, and then filtered through 0.22 μm mesh. RNA was extracted from 350 µL of the nasal swab suspension using RNAios Plus (TaKaRa Bio, Inc., Kusatsu, Japan), according to the manufacturer’s instructions. The cDNA was synthesized using the PrimeScript RT Reagent Kit, according to the manufacturer’s instructions (TaKaRa Bio, Inc., Kusatsu, Japan), and stored at −20 °C.

### 2.3. Detection of BRSV

BRSV was detected by a specific reverse transcription insulated isothermal PCR (RT-iiPCR) assay, which was established and validated in our laboratory. The assay was performed using a POCKITTM device (GeneRadar Biotechnology Corp., Xiamen, China), with default parameters and a reaction program of 95 °C for 58 min. The primer sequences were F: 5′-TGAAAAGYACCCTCATTACAT-3′; R: 5′-CATCACTTGACCTGCTCCAT-3′, and the probe sequences were FAM-TGCAGGGTTATTCATGAATGCATATGGA-BHQ1 (targeting the N gene; fragment length 132bp). Primers and probes were synthesized by Tsingke Biotechnology Corp (Chengdu, China). The amplification was conducted in a 50 μL reaction volume containing 3 μL forward primer (10 µM), 3 μL reverse primer (10 µM), 0.35 μL probe (10 µM), 2 μL of CDNA, 19.65 μL of nuclease-free water, and 24 μL Premix Ex Taq DNA polymerase (5 U/μL) (TaKaRa Biotechnology, Dalian, China).

### 2.4. Amplification of Complete G and F Gene Sequences from Clinical Samples

A pair of primers (G-F: 5′-GAACCATCAACCAATCAAGT-3′; G-R: 5′-CGCCATCCTTATTTGCC-3′) was designed for the amplification of the 1086bp sequence located at nt 4476–5562 in the reference genome USII/S1 (KU159366), which contains the complete G gene sequences. Another pair of primers (F-F: 5′-AGAGAGCACCAAGCAGAGC-3′; F-R: 5′-CATATTTGCAGGGATTTCTTC-3′) was designed for the amplification of the 2273bp sequence located at nt 5263–7526 in the reference genome USII/S1 (KU159366), which contains the complete F gene sequences. Primers were synthesized by Tsingke Biotechnology Co. (Chengdu, China). All amplification products were purified and cloned into the pMD19-T simple vector (TaKaRa Bio, Inc., Kusatsu, Japan) and sequenced Tsingke Biotechnology Co. (Chengdu, China) in both directions.

### 2.5. Genome Amplification

Ten pairs of primers (Table 2) were designed for the amplification of the genome of BRSV according to the complete genome sequence of BRSV in GenBank. Primers were synthesized by Tsingke Biotechnology Co. (Chengdu, China). All amplification products were purified and cloned into the pMD19-T simple vector (TaKaRa Bio, Inc., Kusatsu, Japan) and sequenced Tsingke Biotechnology Co. (Chengdu, China) in both directions. The nucleotide sequence was assembled using SeqMan software (Version 7.0, DNA Star, Madison, WI, USA). Putative ORFs and their corresponding amino acids were predicted using the ORF Finder tool (http://www.ncbi.nlm.nih.gov/gorf/gorf.html), accessed on 12 September 2022.

### 2.6. Sequences, Phylogenetic, and Recombination Analysis

Sequence homology analyses were performed using the MegAlign program of DNASTAR 7.0 software (Version 7.0, DNA Star, Madison, WI, USA). MEGA v.7.0.21 (DNA Star, Madison, WI, USA) was used to perform multiple sequence alignment and to build a neighbor-joining phylogenetic tree with 1000 bootstrap support. The recombination event was assessed using RDP 4.97 and SimPlot software (version 3.5.1, DNA Star, Madison, WI, USA).

## 3. Results

### 3.1. Detection of BRSV

Among 788 clinical samples, 147 samples (18.65%; 0–37%) were detected as BRSV-positive by RT-iiPCR; the positive samples were distributed across 23 cattle farms in 11 provinces. Detailed information on the detection results in different provinces is shown in Table 1 and Figure 1.

### 3.2. Molecular Characterization of the G Gene Sequences

In total, 18 complete G gene sequences (GenBank accession number: OP137017–OP137034) were amplified from clinical samples from 18 farms in eight provinces (Sichuan, Inner Mongolia, Ningxia, Gansu, Shanxi, Henan, Hebei, and Qinghai). The 18 G gene sequences were 792 bp in length, encoding 264 amino acids, and shared 96.6–100% nt identity (94.7–100% aa identity) with each other; moreover, they shared 80.7–95.9% nt identity (69.1–97.1% aa identity) with the known subgroup III strains in GenBank. Interestingly, the 18 G proteins shared seven identical aa mutations, compared with all 55 known subgroup III G proteins in the GenBank **(**Table 3). In addition, compared with the sole Chinese subgroup III strain (DQ strain, MT861050) in GenBank, the 18 G proteins, which were identical in length to some subgroup III strains in GenBank, had 18 continuous nucleotide inserts (nt 5465–5483 in the reference genome USII/S1, KU159366), resulting in 6 amino acid inserts and another 21 identical aa mutations. The details of the amino acid mutations in the strains considered in this study are shown in Table 4.

**Figure 2:**
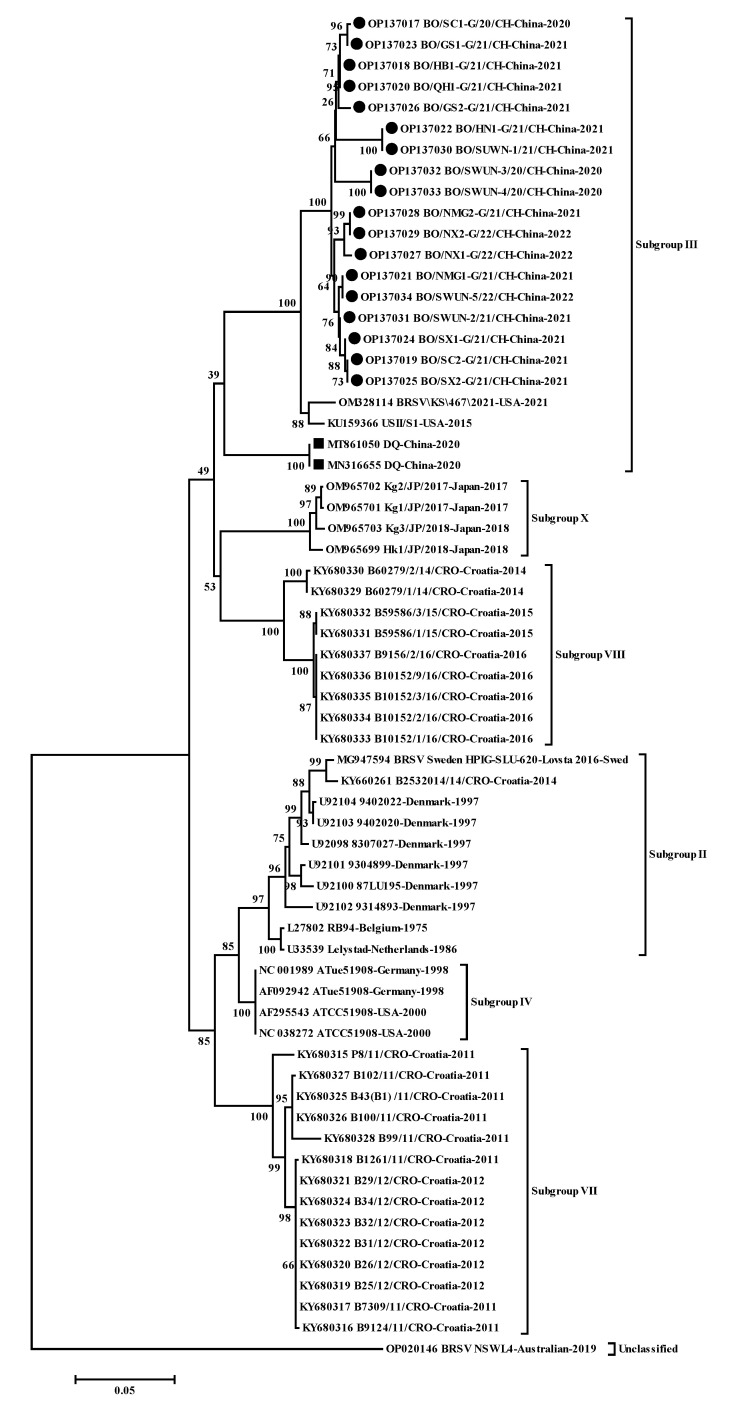
Phylogenetic tree based on the complete nucleotide sequences of G gene. Sequences were compared and clustered by MEGA 7.0 software. The phylogenetic tree was constructed using the neighbor-joining method (1000 replicates). ● represents 18 G gene sequences of BRSV strains from this study, 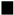 represents the G gene sequences of other strains in China.

A neighbor-joining phylogenetic tree (Figure 2) based on all available complete G nucleotide sequences of BRSV in GenBank indicated that the 18 strains in this study clustered into a large branch with two American subgroup III strains (USII/S1, KU159366, and BRSV\KS\467\2021, OM328114), rather than the sole Chinese strain (DQ strain, subgroup III, MT861050), but were located on an independent small branch.

### 3.3. Molecular Characterization of the F Gene Sequences

In total, 25 complete F gene sequences (GenBank accession number: OP136997–OP137016 and OP137030–OP137034) were amplified from clinical samples in 21 BRSV-positive farms in 10 provinces (Sichuan, Inner Mongolia, Ningxia, Gansu, Shanxi, Henan, Shandong, Yunnan, Hebei, and Qinghai). The 25 complete F sequences were 1710 bp in length, encoding 570 amino acids, and shared 98.5–100% nt identity (97.6–100% aa identity) with each other and 84.8–99.0% nt identity (88.2–99.3% aa identity) with all 22 complete F sequences available in GenBank. Interestingly, compared to other known F proteins in GenBank, the 25 F sequences obtained in this study had 15 continuous nucleotide deletions (nt 5557–5572 in the reference genome USII/S1, KU159366), resulting in 5 amino acid deletions (aa 1–5 in the F protein of reference genome USII/S1, KU159366) in the N-terminal of the F protein due to initiation codon mutation (ATG–ACG); moreover, the 25 F proteins shared additional identical aa mutations (C25G). Furthermore, 12/25 F proteins (GenBank accession number: OP137004–OP137011, OP137014, OP137016, OP137032–OP137033) in this study shared an identical aa mutation (S11C); in addition, 3 F proteins from Gansu Province (GenBank accession number: OP136997–OP136999) shared 10 identical aa mutations (D517Y, V533A, V535A, V537M, V544A, C550W, T552N, R553I, S565N, and I567N) (Figure 2).

**Figure 3:**
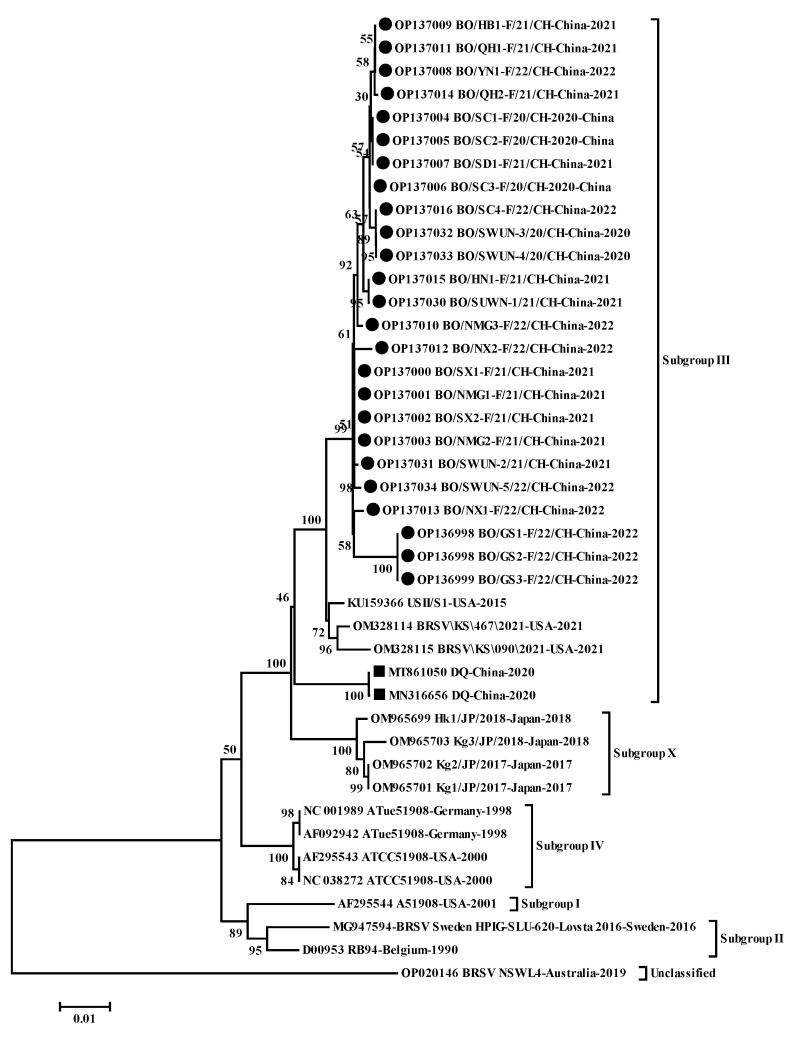
Phylogenetic tree based on the complete nucleotide sequences of F gene. Sequences were compared and clustered by MEGA 7.0 software. The Phylogenetic tree was constructed using the neighbor-joining method (1000 replicates). ● represents 25 F Gene sequences of BRSV strains from this study, 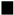 represents the F gene sequences of other isolates in China.

A neighbor-joining phylogenetic tree (Figure 3) based on all the available complete F nucleotide sequences of BRSV in GenBank indicated that the 25 complete F gene sequences clustered into a large branch with two known American subgroup III strains (USII/S1, KU159366, and BRSV\KS\467\2021, OM328114), rather than the sole Chinese strain (DQ strain, subgroup III, MT861050), but were located in an independent small branch.

### 3.4. Genomic Characterization of BRSV Strains

The five nearly complete genome sequences (GenBank accession number: OP137030–OP137034) were successfully amplified from clinical samples obtained in five provinces (Sichuan, Inner Mongolia, Ningxia, Gansu, and Shanxi) and designated as BO/SWUN-1/21/CH (15150bp), BO/SWUN-2/21/CH (15145bp), BO/SWUN-3/20/CH (15142bp), BO/SWUN-4/20/CH (15142bp), and BO/SWUN-5/20/CH (15145bp). The five genome sequences shared 99.2–100% nt identity (99.2–100% aa identity) with each other, and shared 76.8–98.8% nt identity (79.8–99.1% aa identity) with the other thirteen complete genome sequences in GenBank, and shared 95.7–98.2% nt identity (97.1–99.3% aa identity) with the other three subgroup III strains in GenBank (Table 5). With the exception of the identical aa mutations in the G and F proteins described above, the five strains also shared four identical aa mutations, the P protein (A74S), SH protein (N81A), and L protein (F1247C and C954Y), compared to thirteen known complete genome sequences in GenBank (Figure 2).

**Figure 4:**
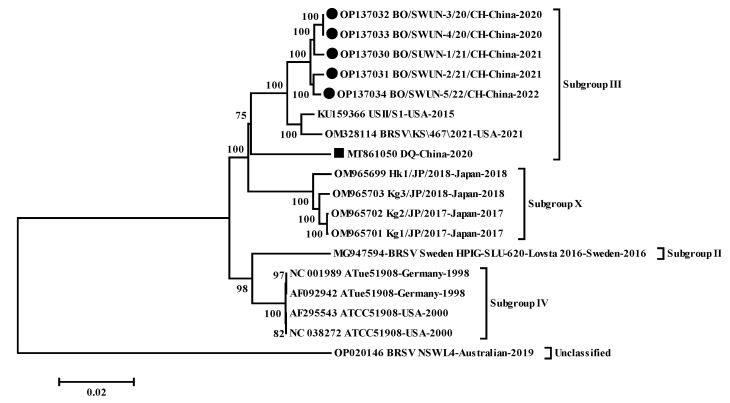
Phylogenetic tree based on the nucleotide sequences of the complete genome was compared and clustered by MEGA 7.0 software. The phylogenetic tree was constructed using the neighbor-joining method (1000 replicates). ● represents 5 complete genome sequences of BRSV strains from this study, 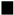 represents the complete genome of other isolates in China.

A neighbor-joining phylogenetic tree (Figure 4) based on all the available nucleotide sequences of the BRSV complete genome in GenBank indicated that five strains clustered into a large branch with two known American subgroups III strains (USII/S1, KU159366, and BRSV\KS\467\2021, OM328114), rather than the sole Chinese strain (DQ strain, subgroup III, MT861050), but were located in an independent small branch. No recombination event was found in the five nearly complete genome sequences.

## 4. Discussion

### 4.1. Prevalence of BRSV in China

In recent years, beef cattle breeding has experienced rapid growth in China, and BRDC has become a major risk in beef cattle. There are multiple causative agents leading to BRDC, among which BRSV is an important pathogen of BRDC; however, the prevalence and molecular characteristics of BRSV in China remain largely unknown. In this study, 18.65% clinical samples of cattle with BRDC were detected as BRSV-positive; the positive samples were distributed in 23 cattle farms across eleven provinces, and the geographical distance between the two farthest provinces was more than 2000 km, which suggests that BRSV has a wide geographical distribution in China. Further, subgroup III strains, which are distributed across America, Turkey, Brazil, and Italy, etc. [3,7,9,23,24], were found to be the dominant BRSV strains in China in this study, and they have unique evolutionary characteristics. The subgroup III strains have caused several outbreaks of respiratory disease in America, which have brought huge economic losses to the cattle industry [7]; therefore, more attention should be paid to BRSV in China’s beef cattle industry. A previous study showed that vaccination was an effective method to prevent BRSV, and there were antigenic differences among different subgroups [2,3]. However, there is still no commercial vaccine for BRSV in China; the results of this study could contribute to BRSV vaccine development in China.

### 4.2. Molecular Characterization of the G Gene Sequences

The G protein was mainly involved in receptor binding and the adsorption process [17] and in the production of antibodies [25]. The G protein consists of three domains: the cytoplasmic domain (1–37aa), the transmembrane domain (38–65aa), and the extracellular domain (66–257aa) [26]. The extracellular domain has a central hydrophobic region (CHR; 158–189aa), which is highly conservative, and 174–185aa in the CHR are immune-dominant [27]. In this study, 18 G proteins shared seven identical aa mutations (Table 3) compared with all subgroup III G proteins in GenBank, and seven aa mutations were located in the extracellular domain. Interestingly, the 18 G proteins had an identical aa mutation (L180S) in the immune-dominant region (174–185aa) of CHR. According to the linear epitopes of the four aa (180aa,183aa, 184aa, and 205aa) of the G protein, BRSV can be divided into four antigen subgroups: A, AB, B, and unclassified [28,29]. Among them, Leu180 and Thr205 were in subgroup A, Leu180 and Ala205 were in subgroup AB, and Pro180 (Ser183 and Pro184) and Ala205 were in subgroup B. Moreover, 180aa mutation may affect the determination of the antigen subgroup of the 18 strains. The effect of the amino acid mutation of the 18 strains on G protein antigenicity needs further study.

### 4.3. Molecular Characterization of the F Gene Sequences

The F protein is involved in the adaptive immune response to stimulate the production of neutralizing antibodies and can promote the entry of viral particles into the host cells and mediate the fusion of infected cells to form syncytium [14,15,16]. The F protein has three hydrophobic peptides, including an amino-terminal signal peptide region (1–26aa), a site for proteolytic cleavage (131–136aa), and a hydrophobic transmembrane anchor sequence (522–549aa). Through the analysis of 25 F proteins in this study, it was found that 25 strains had five amino acid deletions due to initiation codon mutation (ATG–ACG), and an identical aa mutation (C25G), compared with other known strains in GenBank; these mutations were located in the amino-terminal signal peptide region. At present, the effect of the amino-terminal signal peptide region on the detailed function of the F protein is unclear, but the mutation of the initial codon of the F protein of Newcastle Disease Virus (ND), a member of the paramyxovirus family, will affect the virulence of the virus [30]. The effect of initial codon mutation on BRSV’s virulence needs further study. In addition, the hydrophobic transmembrane anchor region (522–549aa) of the F protein involves the anchoring of the F protein to the cell membrane [31]. Interestingly, four amino acid mutations (V533A, V535A, V537M, and V544A) were found in three strains from Gansu Province in this region. Whether the amino acid mutation in this region will affect the anchoring of the F protein and host cells needs further study.

## 5. Conclusions

In conclusion, this study confirmed that BRSV has a wide geographical distribution and that subgroup III strains are the dominant strains in China. The Chinese strains considered in this study showed a unique evolutionary trend based on the phylogenetic analysis of the G, F, and genome sequences, contributing to a better understanding of the prevalence and genetic evolution of BRSV.

## Figures and Tables

**Table 1:** Prevalence of BRSV in nasal swab samples from different provinces or municipalities of China.

Province or Municipality	Number of Farms	Number of Samples	Positive Rate (%)
Gansu	2	40	37.50% (15/40)
Ningxia	2	30	36.67% (11/30)
Sichuan	7	136	36.02% (49/136)
Inner Mongolia	5	58	34.48% (20/58)
Yunnan	1	10	20.00% (2/10)
Heilongjiang	1	12	16.67% (2/12)
Shanxi	6	52	15.38% (8/52)
Hebei	4	41	14.63% (6/41)
Shandong	2	30	13.33% (4/30)
Henan	7	210	13.33% (28/210)
Qinghai	2	16	12.50% (2/16)
Chongqing	6	83	0.00% (0/83)
Sinkiang	2	20	0.00% (0/20)
Jiangsu	1	15	0.00% (0/15)
Anhui	1	15	0.00% (0/15)
Shaanxi	1	10	0.00% (0/10)
Fujian	1	10	0.00% (0/10)
Total	51	788	18.65% (147/788)

**Table 2:** Primers used for RT-PCR amplification of the BRSV genome.

Name	Primer Sequence	Amplified Fragment	Size
F1	ACGCGAAAAAATGCGTATA	1–1175	1176
R1175	GTCTCATTTAGTTTGACCTTGC		
F1028	AATGTCAACCAAATTCCCAC	1028–2966	1938
R2966	TCTTTTCACTTTCTTCATCCC		
F2809	TGTGGCTAGTGCAGGACC	2809–4878	2069
R4878	GGCTGTTATGACGAGTGATG		
F4468	GATAACAAGAGCACATGAAGG	4468–6592	2124
R6592	CCACCCACGATCTGTCCT		
F6381	AAAAGCTAATGTCAAGTAATGTTC	6381–8269	1888
R8269	AGCTCCTGTGATGTCCAATAG		
F8100	TTCCCAGAAAAATACCCTTG	8100–9956	1856
R9956	CAGACAATATAATCAAATCAGCTTC		
F9713	CGGCAAGCAATGGATG	9713–11,598	2281
R11598	CCTAAAGCTTGTGGATCTCTC		
F11351	ATGTTATTTGGTGGTGGAGAC	11,351–12,979	1628
R12979	ATCAGTTATATATCCTTCACCCC		
F12790	CAATAAAACACTTAAGAATAGTCCAC	12,790–14,540	1750
R14540	CTGAATCCTTGTCAATCTTCTTAG		
F14404	AGGTTCTGAGGTTTATTTAGTCC	14,404–15,122	718
R15122	AGAAAAAAAGTATCAAAAACTATCCT		

**Table 3:** Unique Amino acid mutations of G protein sequences in this study compared with all subgroup III strains in GenBank.

G Protein	Amino Acid Mutations						
Amino acid sites	93	100	105	151	180	198	251
Strains in this study	R	Y	R	L	S	L	D
Other subgroup III strains	K	H	S	S	L	P	N

**Table 4:** Amino acid mutations of G protein sequences in this study compare with the sole Chinese subgroup III strain in GenBank.

G Protein	Amino Acid Mutations						
Amino acid sites	24	41	82	90	93	96	100
Strains in this study	L	M	H	S	R	H	Y
Chinese DQ strain	I	L	L	F	K	Y	H
Amino acid sites	105	126	128	147	151	165	168
Strains in this study	R	A	D	S	L	L	S
Chinese DQ strain	S	T	E	P	S	I	L
Amino acid sites	177	180	198	226	229	246	251
Strains in this study	K	S	L	K	P	L	D
Chinese DQ strain	E	L	P	E	L	P	N

**Table 5:** Homology analysis of the 5 genomic sequences in this study with all 13 complete BRSV genomic sequences in GenBank.

Stains	OP137030 BO/SUWN-1/21/CH	OP137031 BO/SUWN-2/21/CH	OP137032 BO/SUWN-3/20/CH	OP137033 BO/SUWN-4/20/CH	OP137034 BO/SUWN-5/22/CH
DQ MT861050	**96.7**	**96.8**	**96.7**	**96.7**	**96.8**
	97	97.1	97	97	97.1
USII/S1 KU159366	**98.7**	**98.8**	**98.7**	**98.7**	**98.8**
	99	99.1	99	99	99.1
BRSV\KS\467 OM328114	**98.6**	**98.7**	**98.6**	**98.6**	**98.7**
	98.8	98.9	98.8	98.8	98.9
HPIG-SLU-620-Lovsta MG947594	**95.4**	**95.5**	**95.5**	**95.5**	**95.5**
	96.4	96.5	96.4	96.4	96.5
Kg3/JP/2018 OM965703	**95.4**	**95.5**	**95.5**	**95.5**	**95.5**
	97	97.1	97	97	97.1
Kg2/JP/2017 OM965702	**96.4**	**96.4**	**96.4**	**96.4**	**96.5**
	97	97.1	97	97	97.1
Kg1/JP/2017 OM965701	**96.4**	**96.4**	**96.4**	**96.4**	**96.5**
	97	97.1	97.1	97.1	97.1
Hk1/JP/2018 OM965699	**96.4**	**96.4**	**96.4**	**96.4**	**96.4**
	96.9	96.9	96.9	96.9	96.9
ATCC51908 NC038272	**96.6**	**96.7**	**96.6**	**96.6**	**96.7**
	97.4	97.5	97.4	97.4	97.5
ATue51908 NC001989	**96.6**	**96.7**	**96.6**	**96.6**	**96.7**
	97.4	97.5	97.4	97.4	97.5
ATCC51908 AF295543	**96.6**	**96.7**	**96.6**	**96.6**	**96.7**
	97.4	97.5	97.4	97.4	97.5
ATue51908 AF092942	**96.6**	**96.7**	**96.6**	**96.6**	**96.7**
	97.4	97.5	97.4	97.4	97.5
BRSV-NSWL4 OP020146	**76.8**	**76.9**	**76.8**	**76.8**	**76.9**
	79.9	79.9	79.8	79.8	79.9

Note: The nucleotide homology of the 5 strains in this study with all 13 known BRSV strains in GenBank were indicated by bold and underlined, and amino acid homology was not shown.

## Data Availability

The data that support the findings of this study are available on request from the corresponding author.
